# CD27^+^ microparticle interactions and immunoregulation of CD4^+^ T lymphocytes

**DOI:** 10.3389/fimmu.2023.1043255

**Published:** 2023-03-09

**Authors:** Léonie Cagnet, Déborah Neyrinck-Leglantier, Marie Tamagne, Lylia Berradhia, Mehdi Khelfa, Sabine Cleophax, France Pirenne, Benoît Vingert

**Affiliations:** ^1^ Univ Paris Est Creteil, INSERM, IMRB, Creteil, France; ^2^ Etablissement Français du Sang, Ivry sur Seine, France; ^3^ Laboratory of Excellence GR-Ex, Paris, France

**Keywords:** microparticles, CD27/CD70, CD4+ TLs, immunomodulation, platelet concentrate, transfusion

## Abstract

**Introduction:**

Aplasia and hematological malignancies are treated with platelet transfusions, which can have major immunomodulatory effects. Platelet concentrates (PCs) contain many immunomodulatory elements, including the platelets themselves, residual leukocytes, extracellular vesicles, such as microparticles (MPs), cytokines and other soluble elements. Two of these components, MPs and a soluble form of CD27 (sCD27), have been shown to play a particularly important role in immune system modulation. The loss of CD27 expression is an irreversible marker of terminal effector CD3^+^ T-lymphocyte (TL) differentiation, and the CD27^+^ MPs present in PCs may maintain CD27 expression on the surface of TLs, and, thus, the activation of these cells.

**Methods:**

In this study, we phenotyped the CD27-expressing MPs present in PCs by microscale flow cytometry and investigated the interaction of these particles with CD4^+^ TLs. We cocultured MPs and PBMCs and determined the origin of the CD27 expressed on the surface of CD4^+^ TLs with the aid of two fluorochromes (BV510 for CD27 originating from MPs and BV786 for cellular CD27).

**Results:**

We showed that the binding of CD27- expressing MPs involved the CD70 molecule, which was also present on these MPs. Finally, the maintenance of CD27 expression on the surface of TLs by sorted CD27^+^ MPs led to activation levels lower than those observed with other types of MPs.

**Discussion:**

These results for CD27-expressing MPs and their CD70-mediated targeting open up new possibilities for immunotherapy based on the use of MPs to maintain a phenotype or to target immune cells, for example. Moreover, decreasing the levels of CD27-expressing MPs in transfused platelets might also increase the chances of success for anti-CD27 monoclonal immunotherapy.

## Introduction

CD27 is a major immune checkpoint constitutively expressed on naive CD3^+^ T lymphocytes (TLs). CD27^-/-^ mice are immunocompetent but have low levels of CD8^+^ TLs (1–3). Effector cell expansion and survival, and the development of memory T cells can be enhanced by CD27 stimulation, which promotes the expression of IL-2, a cytokine essential for the survival of peripheral tissue-resident T cells (1–4). However, CD27 expression decreases after TL activation, leading to irreversible shedding from the cell surface and the formation of soluble CD27 (sCD27) (1, 5, 6). Antigen-presenting cells expressing CD70 trigger the downregulation of CD27 expression after TCR activation. The CD27-CD70 interaction is tightly regulated to prevent overexpression, which might lead to excessive lymphocyte activation. The CD70-CD27 axis is, therefore, a major focus of oncology research (7).

sCD27 has been detected in serum samples from patients with hematological and solid cancers (8–10) and has been implicated in cell activation (11). High plasma sCD27 concentrations are associated with poor patient survival (9). A vesicular form of CD27 has been found in plasma from healthy blood donors (HDs) (12, 13). Two types of extracellular vesicle have been identified: exosomes (small vesicles, 40-100 nm in diameter, derived from intracellular membrane compartments), and larger extracellular vesicles (200-900 nm in diameter, generated by the budding of the cell membrane) known as microparticles (MPs) or ectosomes (14). In this study, we focused exclusively on these larger vesicles budding from the plasma membrane of cells, which we refer to hereafter as MPs. These MPs express many membrane proteins and carry some of the cytoplasm of their cell of origin. They may therefore contain RNA, soluble factors, cytokines, and organelles (14). MPs, which are naturally present in the blood and blood products, play an important role in immunomodulatory processes (14–20).

However, it is difficult to determine the precise role of MPs in immunoregulation due to technical differences between studies, including a lack of MP purification by flow cytometry in some cases. Nevertheless, several functions have been proposed for these vesicles. Heterologous MPs may contain immunoregulatory molecules capable of promoting the lymphoproliferation of CD4^+^ TL, CD8^+^ TL, Treg or Tfh (15, 17, 18) lymphocytes, and modifying the cytokine production profile of lymphocytes or macrophages (17, 19). Heterologous MPs can also alter the differentiation of dendritic cells (19) or Treg (20). Differences in the types of cells from which these MPs originate or in the numbers of these particles present in cultures may also account for functional variability, determining whether MPs activate or inhibit the immune system (15, 20–22). Moreover, heterologous and autologous MPs induce different responses (13, 17), and TCR engagement is one of the factors conditioning MP function (13).

We hypothesized that the transfusion products used to treat hematological malignancies, such as platelet concentrates (PCs) in particular (23, 24), would also contain CD27^+^ MPs, which might have immunomodulatory effects in the patient after transfusion. We tested this hypothesis by determining the levels of CD27^+^ MPs in PCs. CD27 plays an important role in the regulation of CD4^+^ TL responses and CD4^+^ functions during alloimmunization (25–27). We therefore studied the interaction of CD27-expressing MPs with CD4^+^ TLs and its functional effects. In this study, we differentiated between cellular CD27 expression and vesicular CD27 expression through the use of two different fluorochromes during culture. Our findings indicated that the binding of CD27-expressing MPs to CD4^+^ TLs was partly specific. We also performed an *in vitro* assay, to investigate the effect of these CD27-expressing MPs derived from transfused platelet concentrates in the context of targeted immunotherapy based on monoclonal antibodies directed against cellular CD27.

## Materials and methods

### Biological samples

Fresh blood samples were collected from healthy donors (HDs) for PBMC isolation. Blood samples collected into sodium heparin-containing tubes (BD Biosciences, NJ, Franklin Lakes) were provided by the French national blood bank (*Etablissement Français du Sang*, EFS).

MPs were isolated from HD platelet concentrates sampled at the platelet preparation laboratory of the EFS. The platelet concentrates used were either apheresis platelet concentrates (aPCs) or mixed platelet concentrates (mPCs).

None of the HDs had suffered an infection (bacterial, viral, fungal, yeast) or been vaccinated in the 30 days preceding inclusion, and none had received a platelet transfusion. All the participants gave written informed consent.

### MP preparation

MPs were isolated as previously described (13, 15, 26), by differential centrifugation at an initial speed of 3,000 x *g* (4°C, 10 minutes), with the supernatant then centrifuged at 13,000 x *g* (4°C, 10 minutes) for the preparation of a platelet-free supernatant. MPs were concentrated by centrifuging the platelet-free supernatant for 1 hour at 100,000 x *g* (4°C). MPs were resuspended in filter-sterilized (passage through a filter with 0.1 μm pores) PBS for flow cytometry.

### MP phenotyping

MPs were labeled as previously described (13, 15, 26), with fluorochrome-conjugated monoclonal antibodies. Fluorescence was assessed with a 20-parameter LSR Fortessa flow cytometer with a small-particle option (BD Biosciences, San Jose, CA) based on photomultiplier (PMT)-coupled forward scatter (FSC) detection. This mode of detection was used to ensure the optimal detection of MPs with diameters of 200 to 900 nm. The performance of the flow cytometer was checked before each assay. Megamix-Plus FSC and SSC beads (BioCytex, Marseille, France) of known dimensions (beads with diameters ranging from 200 nm to 900 nm) were used to standardize the FSC-PMT parameters and define the MP gate. MPs were labeled with anti-CD27-BV510 and/or anti-CD70-BV786 (BD Biosciences) antibodies and were acquired at low speed and quantified in Trucount tubes (BD Biosciences).

### Assay of CD27-expressing MP binding to PBMCs

MPs were first stained with an anti-CD27- BV510 antibody (BD Biosciences) at 4°C for 30 minutes and then washed by centrifugation for 1 hour at 100,000 x *g* and 4°C, to eliminate the antibodies that had not bound to MPs. MPs were resuspended in filter-sterilized medium (passage through a filter with 0.1 μm pores) and the CD27^+^ MPs present in the MP preparation were counted.

PBMCs were isolated by Ficoll density gradient centrifugation. We cultured 2 x 10^4^ PBMCs for 18 hours with quantified CD27-expressing MPs. We added various amounts of the bulk MP preparation to the culture medium (filter-sterilized, passage through a filter with 0.1 µm pores) such that we obtained ratios of PBMCs to CD27-expressing MPs of 1:1, 1:10 and 1:20 (PBMC: CD27-expressing MPs).

The culture medium consisted of RPMI 1640 supplemented with 5% FBS (Dutscher, Bernolsheim, France), 2 mM L-glutamine, 100 µg/ml penicillin/streptomycin, MEM non-essential amino acids solution (1X), and 1 mM sodium pyruvate (all from Life Technologies, Carlsbad, CA).

Following coculture, the PBMCs were harvested for flow cytometry to assess their co-expression of CD27 from MPs (BV510-labeled). PBMCs were stained with anti-CD4-APC-H7 and anti-CD3-PE (BD Biosciences) antibodies. After staining (4°C, 30 minutes), the cells were washed twice with PBS.

### Assay of CD27 cellular expression after CD27-expressing MP binding

For this assay, before culture with BV510-labeled CD27-expressing MPs (as previously described in “Assay of CD27^+^ MP binding to PBMCs” section), the PBMCs were stained with anti-CD27-BV786 antibody, to make it possible to distinguish cellular CD27 from the CD27 expressed by MPs. After staining (4°C, 30 minutes), the cells were washed twice with PBS.

Following coculture, the PBMCs were harvested for flow cytometry to assess their co-expression of CD27 from MPs (BV510-labeled) and cellular CD27 (BV786-labeled). PBMCs were stained with anti-CD4-APC-H7 and anti-CD3-PE (BD Biosciences) antibodies. After staining (4°C, 30 minutes), the cells were washed twice with PBS.

### Assay of CD27^+^ MP binding to PBMCs after the CD70 treatment of PBMC or MPs

We investigated the role of CD70 in the association of CD27^+^ MPs with CD4^+^ TLs, by staining MPs with anti-CD70 antibody (BV786-labeled) before culture (10 μg, BD Biosciences). After staining (4°C, 30 minutes), the MPs were washed by centrifugation for 1 hour at 100,000 x *g* (4°C) to eliminate the antibodies that had not bound to MPs. PBMCs were cultured for 18 hours in the conditions described in the “Assay of CD27^+^ MP binding to PBMCs” section. PBMCs were stained with anti-CD4-APC-H7 and anti-CD3-PE (BD Biosciences) antibodies for 30 minutes at 4°C and were then washed twice with PBS.

We investigated the role of cellular CD70 in the interaction of CD4^+^ TLs with CD27^+^ MPs, by staining PBMCs with an anti-CD70 antibody (BV786-labeled) before culture (4°C, 30 minutes, 10 μg, BD Biosciences). PBMCs were washed by centrifugation (4°C, 600 x *g*) to eliminate the anti-CD70 antibodies that had not bound to PBMCs. Cells were cultured for 18 hours in the conditions described in the “Assay of CD27^+^ MP binding to PBMCs” section. PBMCs were stained with anti-CD4-APC-H7 and anti-CD3-PE (BD Biosciences) antibodies for 30 minutes at 4°C and were then washed twice with PBS.

### CD27^+^ MP sorting by flow cytometry

For proliferation assays, CD27^+^ MPs were sorted with a MoFlo Astrios flow cytometer (Beckman Coulter, Brea, CA) equipped with a PMT-FSC detector, as previously described (13, 15). Flow cytometer performance was assessed with Megamix-Plus FSC and SSC beads (BioCytex) before the MP sorting experiments. MPs were labeled with anti-CD27-BV510 (BD Biosciences) antibody (4°C, 30 minutes). We purified CD27^+^ MPs with diameters between 200 and 900 nm. We used a commercial kit to check for the absence of endotoxin in the sorted CD27^+^ MP preparations (*In vivo*gen, San Diego, CA).

### Proliferation of CD4^+^ TLs after interaction with sorted CD27^+^ MPs

We assessed CD4^+^ T-cell proliferation, as previously described (13, 15). CFSE-labeled PBMCs were treated in culture medium for six days with sorted CD27^+^ MPs at ratios of 2000:1, 200:1, 20:1, 1:1 and 1:10 (PBMC: sorted CD27^+^ MPs). Lymphoproliferation was measured by flow cytometry analyses of CD4^+^ TLs with anti-CD4-APC-H7 and anti-CD3-PE (BD Biosciences) antibodies. Aqua Live/Dead viability dye (Thermo Fisher Scientific, MA, Waltham) was added to exclude dead cells. Lymphoproliferation was normalized between donors. For each HD tested, a proliferation index of 1 was assigned to the lymphoproliferation observed in the absence of MPs. Lymphoproliferation is expressed proportionally, as the fold-induction relative to lymphoproliferation in the absence of MPs.

### MP-CD4^+^ T cell competition assay for anti-CD27 antibody binding

For this assay, the samples of CD27-expressing MPs to be used in the competition test were first subjected to counting by flow cytometry in Trucount tubes, to determine the numbers of CD27^+^ MPs present in the samples tested.

Competition assays were performed with heterologous PBMCs, various amounts of bulk MP preparation to give different ratios of PBMCs to CD27-expressing MPs (based on the counts obtained), and an anti-CD27 antibody (BV510-labeled). The ratios tested were (PBMC: CD27-expressing MPs) 10:1, 1:1, 1:10, and 1:20. The controls used were cultures either without cells or without MPs.

After 60 min of culture at 37°C, cells were isolated for flow cytometry-based determinations of BV510-labeled anti-CD27 antibody binding to CD4^+^ TLs. CD4^+^ TLs were identified with anti-CD3 BUV737 and anti-CD4 PE-CF594 antibodies (BD Biosciences).

### Flow cytometry analysis

For phenotyping, fluorescence was assessed on an LSR Fortessa flow cytometer (BD Biosciences), and the flow cytometry data were analyzed with FlowJo software (v.10.8.1, Ashland, OR).

### Statistical analysis

All analyses were performed with Prism 6.07 software (GraphPad Software, La Jolla, CA). Only significant differences between groups (*p*<0.05) are indicated on the data plots.

## Results

### CD27 expression on MPs from platelet concentrates

**Figure 1:**
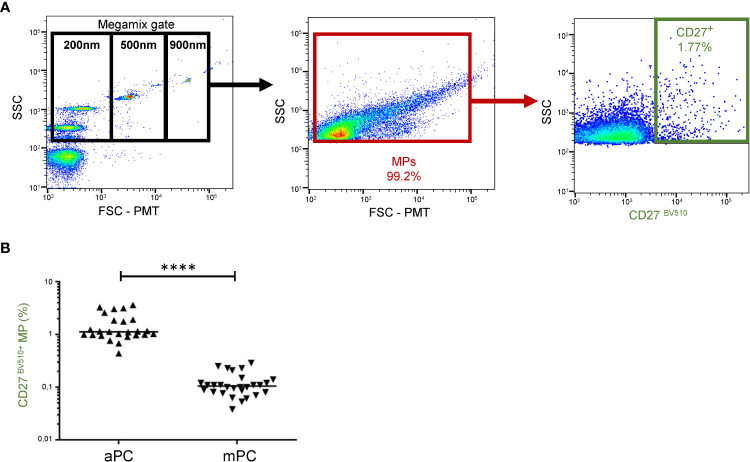
CD27 expression on MPs from platelet concentrates. **(A)** Example of the gating strategy used in the phenotyping of MPs from HDs. On the left, a dot plot showing the settings, based on Megamix fluorescent beads, for the differentiation of three particle sizes: 200, 500 and 900 nm in diameter. **(B)** CD27 phenotyping was performed with a anti-CD27-BV510 antibody on apheresis platelet concentrates (aPCs) from 25 HDs in two experiments and on mixed platelet concentrate (mPCs) pools for 28 HDs in two experiments. The horizontal bar indicates the median value. The *P* values were obtained in Mann-Whitney *post hoc* tests: ****P < 0.001.

CD27 expression on MPs from platelet concentrates was measured by flow cytometry (**Figure 1A**). In aPCs, 1.52 ± 0.90% of the MPs expressed CD27. In mPCs, the percentage of MPs expressing CD27 was significantly lower, at only 0.12 ± 0.06% (*P*<0.001). Moreover, the frequency of CD27 expression on MPs was highly variable, ranging from 0.44 to 3.63% for aPCs and from 0.04-0.29% for mPCs (**Figure 1B**).

### Interaction of CD27-expressing MPs with CD4^+^ TLs

**Figure 2:**
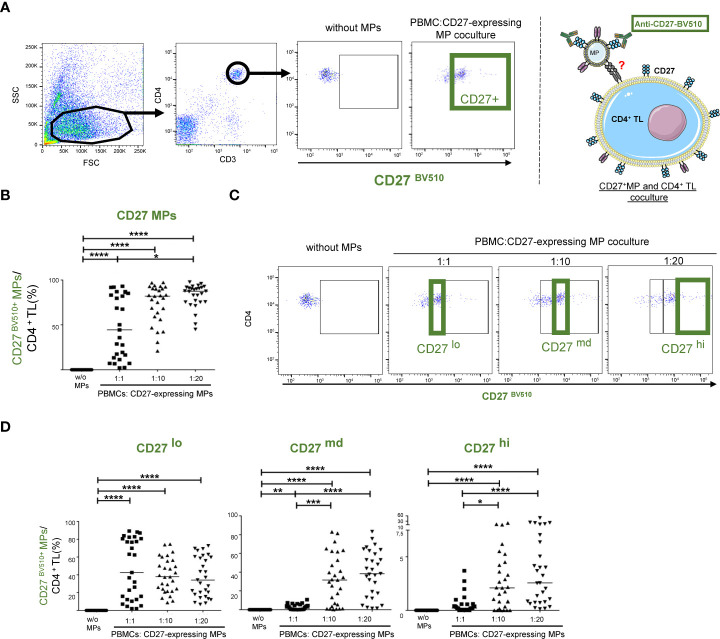
CD27^+^ MP expression on CD4^+^ TLs after the coculture of MPs and PBMCs. Before culture, MPs were stained with a BV510-labeled anti-CD27 antibody. Samples from 29 HDs were used in 10 coculture experiments. **(A)** Example of the gating strategy used to assess the interaction of CD27-expressing MPs with CD4^+^ TLs (PBMCs were stained with anti-CD4 and anti-CD3 antibodies; CD4^+^ TLs were defined as having a CD3^+^CD4^+^ phenotype), and schematic representation of CD27^+^ MP binding to CD4^+^ TLs at 1:1 ratio (PBMCs: CD27-expressing MPs). PBMCs were cocultured for 18 hours with known numbers of CD27-expressing MPs, at ratios of 1:1, 1:10 and 1:20 (PBMCs: CD27-expressing MPs). **(B)** Percentage of CD4^+^ TLs expressing CD27^+^ from MPs at different coculture ratios (PBMCs: CD27-expressing MPs). Horizontal bars indicate the median value. **(C, D)** Percentage of CD27^+^ MP expression in the different gates based on the relocation of the MIF. Horizontal bars indicate the median value. *P* values were obtained in Kruskal-Wallis and Dunn’s *post hoc* tests: ****P < 0.001, ***P < 0.005, **P < 0.01, *P < 0.05.

PBMCs were cultured with MPs as a means of investigating the interaction of CD27-expressing MPs with CD4^+^ TLs. This interaction was assessed by labeling the CD27-expressing MPs with an anti-CD27 BV510 antibody (**Figure 2A**).

The variation of CD27 expression levels among MPs in platelet concentrates was mimicked by coculturing CD4^+^ TLs with different ratios of MPs to cells (**Figure 2B**). At higher PMBC : CD27-expressing MPs ratios, significant association with CD3^+^CD4^+^ T lymphocytes was observed (**Figure 2B**) (*P*<0.001). At a 1:1 (PBMCs: CD27-expressing MPs) ratio, associations were observed for 47.8 ± 34.8% of the CD4^+^ TLs. The frequency of these interactions was highly heterogeneous, occurring with 2.11 to 93% of CD4^+^ TLs (**Figure 2B**). At a ratio of 1:10, a mean of 74.5 ± 21.6% of the CD4^+^ TLs interacted with the CD27-expressing MPs. The frequency of interaction was again highly heterogeneous, ranging from 21 to 97.3% (**Figure 2B**). Finally, at the highest density of CD27-expressing MPs tested (1:20 ratio), a mean of 82.8 ± 13.3% of the CD4^+^ TLs interacted with the CD27-expressing MPs, with the frequency of interaction ranging from 44.9 to 97.7% (**Figure 2B**).

We identified three populations interacting with CD4^+^ TLs, defined according to the intensity of CD27^BV510^ expression on the CD4^+^ TLs: CD27^lo^ (MFI < 5 x 10^3^), CD27^md^ (MFI, [5 x 10^3^-10^4^]) and CD27^hi^ (MFI>10^4^) (**Figure 2C**). The CD27 BV510^lo^ population was present at similar frequencies for all ratios used (**Figure 2D**). By contrast, the CD27^md^ population was present in the culture at a 1:1 ratio, but its levels were significantly higher for the 1:10 and 1:20 ratios (*P*<0.001), with no difference between its frequencies at these two higher ratios (**Figure 2D**). For this CD27^md^ population, mean interaction rates of 30.7% and 38.1% were obtained at the 1:10 and 1:20 ratios, respectively (**Figure 2D**). Similar results were obtained for the CD27^hi^ population, with mean interaction rates of 3.2% and 7% for the 1:10 and 1:20 ratios, respectively (**Figure 2D**).

### Amplification of the CD27^+^ cell phenotype after binding to CD27-expressing MPs

**Figure 3:**
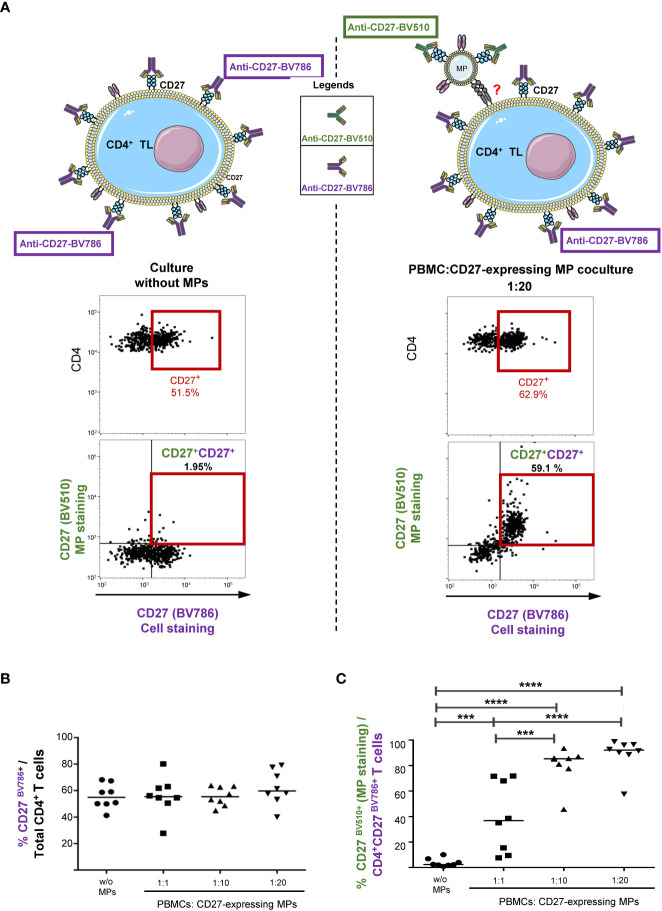
Impact of CD27^+^ MP binding on the pre-existing CD27 phenotype of the CD4^+^ TLs. **(A)** Schematic representation of the assay, and example of the gating strategy used to determine the CD27 expression of CD4^+^ TLs (PBMCs were stained with anti-CD4 and anti-CD3 antibodies; CD4^+^ TLs were defined as having a CD3^+^CD4^+^ phenotype). Cocultures were assessed after 18 hours for eight HDs in two experiments. Before culture, MPs were stained with a anti-CD27-BV510 antibody and PBMCs were stained with a anti-CD27-BV786 antibody. **(B)** Percentage CD27^BV786+^ CD4^+^ TLs with and without coculture with MPs. **(C)** Percentage of CD27^BV510+^ CD4^+^CD27^BV786+^ TLs with and without coculture with MPs. Horizontal bars indicate the median value. *P* values were obtained in Kruskal-Wallis and Dunn’s *post hoc* tests: ****P < 0.001, ***P < 0.005.

We investigated the effect of the binding of CD27-expressing MPs on the pre-existing CD27 phenotype of CD4^+^ TLs, by labeling CD4^+^ TLs with a BV786-conjugated anti-CD27 antibody (CD27^BV786+^) before culture with MPs (CD27^BV510+^). CD27 expression was studied in the presence and absence of MPs in order to highlight the fact that after coculture, CD27 expression on CD4^+^ TLs is either due to cellular and/or to the binding of CD27 expressing MPs to CD4^+^ TLs (**Figure 3A**).

In the absence of MPs, 55.8 ± 9.3% of CD4^+^ TLs displayed CD27^BV786^ expression (**Figure 3B**). In coculture with MPs, the mean levels of CD27^BV786^ expression were similar for all PBMC : CD27-expressing MP ratios (for a 1:1 ratio, 56.1 ± 14.6%; for a 1:10 ratio, 55.8 ± 7.1%; and for a 1:20 ratio, 62.3 ± 13.1%) (**Figure 3B**).

During coculture with MPs, very little binding of CD27 ^BV510+^ MPs to CD4^+^CD27^neg^ TLs was observed (**Figure 3A**, right panel). Almost all the interactions observed involved CD4^+^ TLs that already had a CD27^+^ phenotype (**Figure 3A**, right panel). Thus, the CD27-expressing MPs bound to 39.7 ± 27.8% of CD4^+^CD27^BV786^ TLs for cocultures at a 1:1 ratio, 80.3 ± 14.6% for the 1:10 ratio, and 89.1 ± 13.2% for the 1:20 ratio (**Figure 3C**).

### Role of CD70 in the interaction between CD27-expressing MPs and CD4^+^ TLs

As CD4^+^ CD27^neg^ TLs did not interact with CD27-expressing MPs, we hypothesized that the observed binding might be due to the co-expression of another immune regulatory molecule, as MPs can co-express several immune checkpoints (14). The CD27-expressing MPs appeared to bind only CD4^+^ TLs that were already CD27^+^ (**Figure 3A**). We therefore hypothesized that co-expression of the CD27 ligand CD70 might be involved.

**Figure 4:**
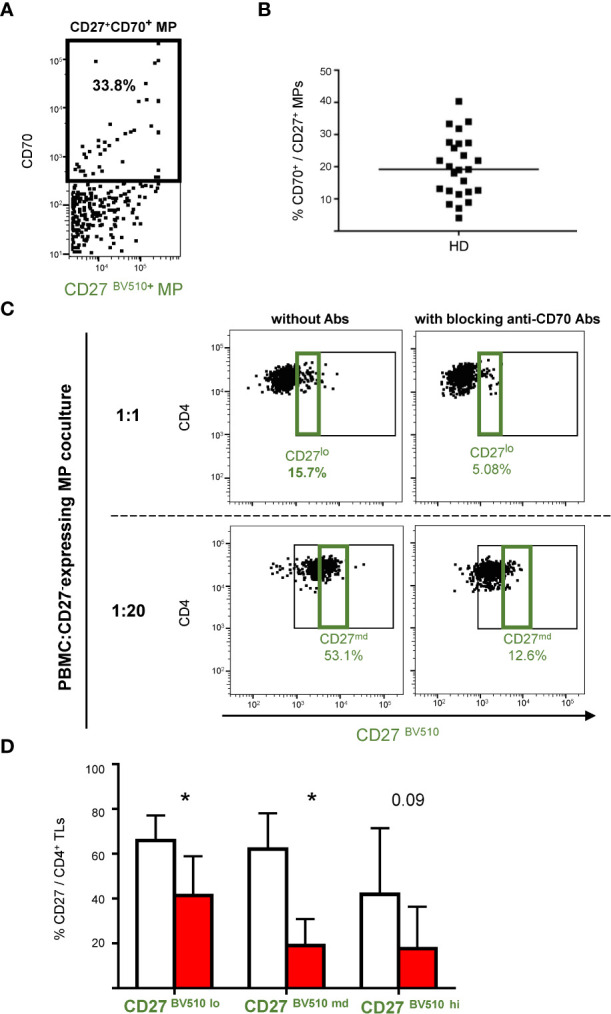
CD27^+^ MP binding to CD4^+^ T cells after coculture with CD27/CD70-labeled MPs. **(A)** Example of the gating used for phenotyping CD27^+^ MPs from one HD for the CD70 receptor. Only MPs expressing CD27 are represented on this plot. Phenotyping was performed on MPs stained with a anti-CD27-BV510 antibody from 25 HDs in two experiments. **(B)** Percentage of CD27^+^ MPs expressing CD70. The horizontal bar indicates the median. **(C)** Example of the gating strategy used to highlight the expression of CD27 from MPs on the surface of CD4^+^ TLs (PBMCs were stained with anti-CD4 and anti-CD3 antibodies; CD4^+^ TLs were defined as having a CD3^+^CD4^+^ phenotype; only CD4^+^ TLs are presented), in the presence and absence of blocking anti-CD70 antibody. PBMCs were cocultured for 18 hours with known numbers of CD27-expressing MPs at ratios of 1:1, 1:10 and 1:20 (PBMCs: CD27-expressing MPs). Cocultures were assessed for seven HDs in four experiments. **(D)** Percentage of CD4^+^ TLs expressing CD27^+^ from MPs at different coculture ratios (PBMCs: CD27-expressing MPs) in the presence and absence of blocking anti-CD70 antibody. The percentage of CD4^+^CD27^lo^ TLs was determined for the 1:1 ratio (PBMCs: CD27-expressing MPs); the percentage of CD4^+^CD27^md^ TLs was determined for the 1:10 ratio, and the percentage of CD4^+^CD27^hi^ TLs was determined for the 1:20 ratio, with (■) and without (□) blocking anti-CD70 antibody. The *P* values presented were obtained in *t* tests: * *P* < 0.05.

Flow cytometry analyses showed that CD70 was expressed on a mean of 19.9 ± 9.4% of CD27-expressing MPs (**Figures 4A, B**). We investigated the functional role of CD70 in the CD27^+^MP/CD4^+^TL interaction. To this end, MPs were co-stained with an antibody directed against CD70, to block the CD70 on CD27-expressing MPs potentially used for binding to cellular CD27.

The binding of CD27-expressing MPs was measured in the presence and absence of this anti-CD70 antibody, for all three PBMC: CD27-expressing MP coculture ratios. The CD4^+^ TLs had a lower CD27^BV510^ fluorescence intensity in the presence of this antibody (**Figure 4C**). At a coculture ratio of 1:1, the proportion of CD27^lo^ CD4^+^ TLs decreased significantly, from 65.9 ± 9.9% to 41.3 ± 17.6%, in the presence of the blocking antibody (*P*<0.05) (**Figure 4D**). Likewise, at a coculture ratio of 1:10, the proportion of CD27^md^ CD4^+^ TLs decreased significantly, from 62.1 ± 13.8% to 19.1 ± 11.8%, in the presence of the blocking antibody (*P*<0.05) (**Figure 4D**). Finally, a similar trend was observed at the 1:20 ratio, with the proportion of CD27^hi^CD4^+^ TLs decreasing from 41.9 ± 25.6% to 17.7 ± 18.6% in the presence of the blocking antibody (*P*<0.05) (**Figure 4D**).

We decided, to study the blockade of cellular CD70. To this end, before coculture with CD27-expressing MPs, we labeled the CD4^+^ TLs with an anti-CD70 antibody, to block the binding of the cellular CD70 to the CD27 expressed by MPs.

We found that CD70 was expressed on 2.14 ± 0.98% of CD4^+^ TLs (**Supplementary Figures 1A, B**). We found no significant difference in percentages of CD4^+^CD27^+^ TLs, regardless of the BV510 fluorescence intensity between culture conditions with or without blocking anti-CD70 antibody (**Supplementary Figures 1C, D**).

### CD4^+^ TL activation driven by CD27-expressing MPs

The functional properties of these MPs, and their interactions with TLs in particular, were unknown. We investigated these properties by studying the effects of these CD27-expressing MPs on cytokine production by CD4^+^ TLs, which is known to play a major role in alloimmune responses, including anti-platelet responses in particular (23–25).

We used an intracellular cytokine staining (ICS) assay to study the direct activation of CD4^+^ TLs after binding to CD27-expressing MPs. We studied the production of IL-2, IFNγ and TNFα by CD4^+^ TLs after culture for 18 h. The negative controls were PBMCs not cultured with MPs (Data not shown). The positive controls were PBMCs stimulated with the staphylococcal enterotoxin B superantigen (1 μg/ml, List Biological Laboratories, Inc, Campbell, CA) (Data not shown). None of these cytokines was produced in significant quantities after the coculture of CD4^+^ TLs with CD27-expressing MPs (**Supplemental Figure 2**).

**Figure 5:**
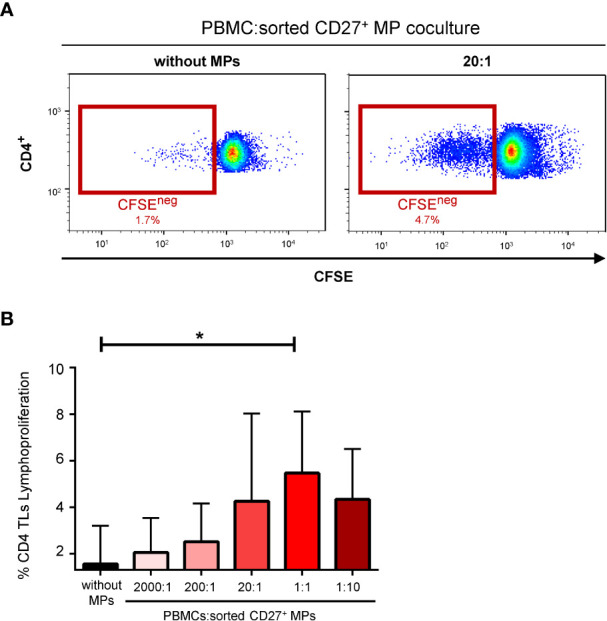
Effect of CD27^+^ MPs on CD4^+^ TL lymphoproliferation. **(A)** Representative FACS plots measuring the proliferation of CD4^+^ TLs over a period of six days in seven coculture experiments corresponding to seven HDs (PBMCs were stained with anti-CD4 and anti-CD3 antibodies; CD4^+^ TLs were defined as having a CD3^+^CD4^+^ phenotype). This functional assay was also performed with various proportions of sorted CD27^+^ MPs. **(B)** Percentage of CD4^+^ TLs proliferating, for different ratios of PBMCs:sorted CD27^+^ MPs. The *P* values were obtained in ANOVA and Kruskal-Wallis *post hoc* tests: *P < 0.05.

Strong stimulation of the proliferation of CD4^+^ TLs by MPs has been reported (14). We therefore performed lymphoproliferation experiments with sorted CD27^+^ MPs. The CD4^+^ TLs proliferated in the presence of CD27^+^ MPs sorted by flow cytometry (**Figure 5A**). This functional assay was also performed with various proportions of sorted CD27^+^ MPs. Lymphoproliferation rates were highest at a coculture ratio of 1:1, at which 4.6 ± 3.2% of CD4^+^ TLs proliferated (*P*<0.05) (**Figure 5B**).

### CD27-expressing MPs from platelet concentrates can compete with cellular targets during immunotherapies based on monoclonal antibodies

**Figure 6:**
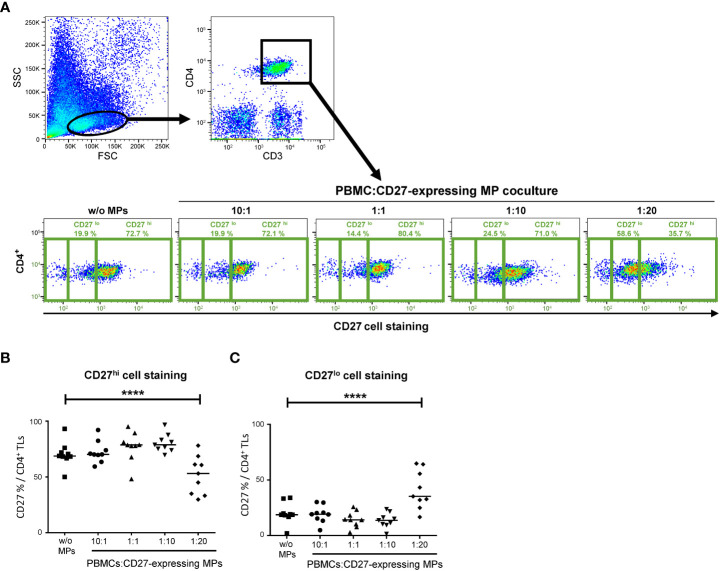
Assays of competition between CD27^+^ MPs and PBMCs for CD27 staining. **(A)** Example of the gating used for assessing the CD27 expression of CD4^+^ T cells cocultured (PBMCs were stained with anti-CD4 and anti-CD3 antibodies; CD4^+^ TLs were defined as having a CD3^+^CD4^+^ phenotype), at various ratios, with MPs from one HD. PBMCs were incubated with 1) different amounts of MP preparations containing known numbers of CD27-expressing MPs, to obtain different ratios of cells to CD27-expressing MPs and 2) an anti-CD27 antibody (labeled with BV510). Phenotyping was performed on CD4^+^ TLs from 9 HDs in 6 experiments. **(B)** Percentage of CD4^+^ LTs with high levels of CD27 staining (MFI >8 x 10^2^). **(C)** Percentage of CD4^+^ LTs with low levels of CD27 staining (0.5 x 10^2^<MFI<8 x 10^2^). The horizontal bar indicates the median. The *P* values were obtained in ANOVA and Kruskal-Wallis *post hoc* tests: ****P < 0.001.

It has been suggested that MPs are involved in resistance to anti-PD1 immunotherapies (28–34). CD27-expressing MPs supplied by the transfusion of platelet concentrates could, therefore, be involved in the failure of anti-CD27 immunotherapy. The mechanism of action of MPs has not yet been determined (28–34), but we suggest that MPs present in transfusion products may compete with cellular targets. We tested this hypothesis by performing assays of *in vitro* competition between MPs and cells (**Figure 6**), with the same ratios of CD27-expressing MPs to cells as above.

A decrease in the intensity of CD27 cell labeling was observed when TLs were cocultured with CD27-expressing MPs in competition assays. This decrease in MFI for the bright CD27 (CD27^hi^) signal was significant for the coculture ratio of 1:20 relative to a culture without MPs (51.6 ± 16.9% vs. 70.4 ± 11.1%, respectively, *P*<0.001) (**Figures 6A, B**). This decrease in MFI for the CD27^hi^ signal was accompanied by a significant increase in the CD27^lo^ signal for a coculture ratio (PBMC: CD27-expressing MP) of 1:20 relative to a culture without MPs (41.1 ± 17.1% vs. 20.1 ± 9.4%) (**Figures 6A, C**).

## Discussion

Immune checkpoint targeting with monoclonal antibodies is a widely used immunotherapy approach for limiting tumor growth. The presence of microvesicles/MPs expressing PD1/PD-L1 is often associated with treatment responsiveness (28–34). However, the possibility of these MPs being transferred into the body in a context of polytransfusion is rarely considered.

Immunotherapy strategies aiming to eradicate tumors by targeting the CD70-CD27 axis, mostly targeting CD70, have been explored principally in the context of acute myeloid leukemia (7). However, vesicular forms of CD70 or CD27 present in transfusion products must be taken into account, because many patients undergo multiple transfusions, with platelet concentrates in particular (23, 24). In this study, we focused on CD27^+^ MPs from platelet concentrates, a previously unconsidered vesicular supply of CD27.

CD27^+^ MPs have recently been detected in plasma (12). We confirmed their presence in plasma in a study on a new method for identifying plasma MPs without ultracentrifugation (13). In HDs, CD27^+^ MPs account for 4.3 ± 1.0% of the MPs present in plasma, *i.e.* 1.3 x 10^5^ ± 0.4 x 10^5^ CD27^+^ MPs/ml of plasma. The presence of these MPs in platelet concentrates would therefore be expected. Nevertheless, the proportion of MPs expressing CD27 was lower in PCs than in plasma, regardless of the type of PC considered. Given the very large difference in CD27 expression between aPCs and mPCs, we hypothesized that production processes might be responsible for these differences. We found that filtration units, such as those used for leukoreduction, can retain some MPs (data not shown). We also observed significant differences in expression between PCs from different donors; a similar heterogeneity has also been reported for HD plasma (15, 26). Its origin is thought to be multifactorial (14).

CD27 is found principally on the surface of naïve cells and is lost with cell activation (1, 2). We therefore hypothesized that the concentration of CD27^+^ MPs in PCs might reflect cell activation. However, CD27 may originate from several cell types, including T cells, B cells, NK cells and hematopoietic cells (1, 2). These differences in cellular origin may influence the level of CD27 expression on the surface of MPs.

The loss of CD27 expression is typically irreversible in effector TLs, but we hypothesized that the CD27^+^ MPs present in PCs might conserve their CD27 expression on the surface of TLs after interactions. Our data for PBMC: CD27-expressing MPs cocultures confirmed that these CD27^+^ MPs interacted with CD4^+^ TLs. One important feature of this interaction is the correlation between the number of CD27 molecules derived from MPs on the surface of CD4^+^ TLs, and the number of CD27-expressing MPs in culture. However, binding appeared to reach saturation at a coculture ratio of 1:20 (PBMCs: CD27-expressing MPs).

Our results suggest that transfusion with a single platelet concentrate would lead to the transfer of a mean of 8 x 10^7^ CD27^+^ MPs, and a maximum of up to 54 x 10^7^ CD27^+^ MPs. Patient management regimens involving polytransfusion can, therefore, rapidly increase the total numbers of exogenous MPs transferred into the body of the patient, but also the number of CD27^+^ MPs received. Studies in a mouse model will be required to determine whether these 1:20 ratios are compatible with platelet polytransfusions. The use of such a model would also make it possible to determine whether such ratios can be reached in secondary lymphoid organs.

The observed interactions almost exclusively involved cells that already expressed CD27. We hypothesized that this interaction involves a ligand/receptor pair, probably CD27 and its ligand, CD70. We showed in a previous study that MPs derived from a transfusion product (pRBC) co-expressed several immune checkpoints (15). A blockade of the CD70 expressed by these MPs partially inhibited binding, suggesting that other ligand/receptor pairs must also be involved in these interactions. This result suggests that other ligand/receptor pairs must also be involved in these interactions. However, regardless of the ligand/receptor pair involved, the partial neutralization of the binding of MPs co-expressing CD27 and CD70 demonstrates that the binding of these particles can be directed.

**Figure 7:**
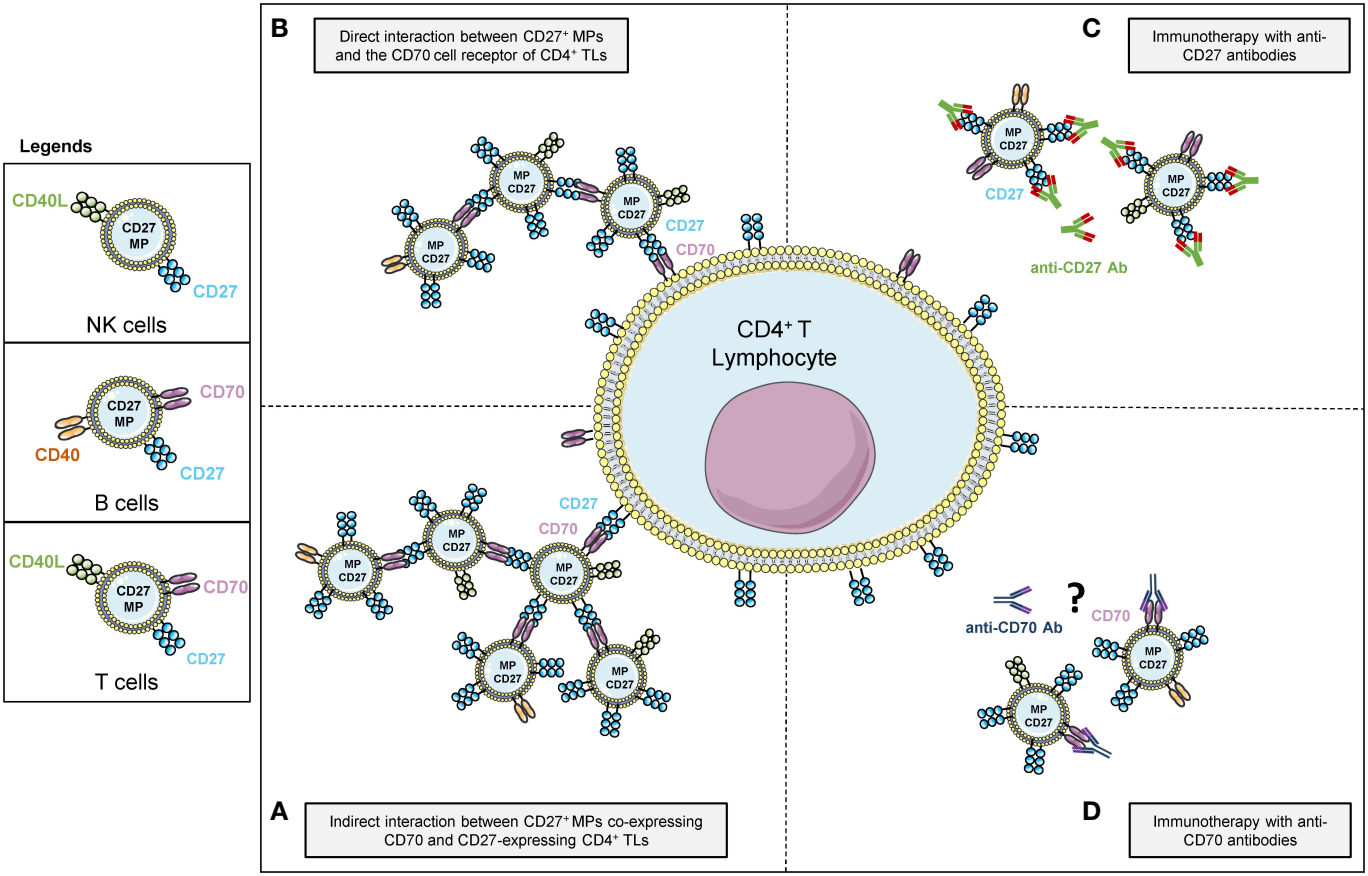
Schematic representation of the mechanism of action of vesicular forms of CD27 of different cellular origins against CD4^+^ TLs **(A)** Mode of agglutination attachment of MPs to CD4^+^ TLs *via* the binding of cellular CD27 and vesicular CD70 receptors. **(B)** Mode of agglutination attachment of CD27^+^ MPs to CD4^+^ TLs *via* the binding of cellular CD70 and vesicular CD27 receptors. **(C)** Effect of competition between CD27^+^ MPs and CD4^+^ TLs for binding to monoclonal anti-CD27 antibodies. **(D)** Hypothesis concerning the effect of monoclonal anti-CD70 antibodies on the interaction between CD27^+^ MPs and CD4^+^ TLs.

The immune cell origin and activation molecules of MPs may drive functional modifications of the immune system. Several groups have reported functional differences between MPs of different cellular origins (18–21). For example, the CD40/CD40L receptor/ligand pair on MPs has also been implicated in T-cell responses (35) and has been detected on blood MPs (15). Indeed, CD40L is found on TLs and NK cells, whereas CD40 originates from B lymphocytes. Thus, if the MPs originate from TLs, their phenotype may be CD27^+^CD70^+^CD40L^+^, whereas, if they originate from B cells, they will have a CD27^+^CD70^+^CD40^+^ phenotype. The cellular origin of the CD27 on MPs is therefore important, as summarized in **Figure 7**.

The functional benefits of heterologous MPs were found to be totally independent of antigenic stimulation, as previously described for TGFβ^+^ MPs and unsorted MPs from red blood cell concentrates (15). The origin of this functionality remains unclear, but we have recently shown a possible association with sCD27 in cocultures of MPs and autologous CD4^+^ TLs (13).

The possible impact of providing heterologous CD27^+^ MPs *via* platelet transfusion remains unclear, given the low levels of lymphoproliferation observed with CD27-expressing MPs. Nevertheless, as suggested by our competition assays, the presence of MPs should be taken into account in patients prescribed monoclonal immunotherapy, for malignant cancers, for example (**Figure 7C**).

Indeed, as CD27 stimulation can enhance TL function (1, 2), blockade of the PD1/PDL1 axis in combination with agonistic CD27 monoclonal antibody treatment has been proposed as a means of enhancing the expansion and function of the CD8^+^ cytotoxic TL population (36). However, as already reported for the soluble forms of PDL1 (37), interactions with sCD27 or CD27^+^ MPs may occur in immunotherapy of this type. Similarly, sCD27, which is strongly expressed in cancers (8–10), might interact with these CD27^+^CD70^+^ expressing MPs, thereby canceling out the benefits of transfusion.

The CD70/CD27 axis is a major focus of treatment strategies in oncology, and the effects of monoclonal anti-CD70 antibodies may also be attenuated by transfusions of PCs rich in CD27^+^CD70^+^ co-expressing MPs (7) (**Figure 7D**).

MPs are often disregarded as cellular waste, but rather than being passive elements, they are actually involved in intercellular communication (14, 16). We show here that MP interactions can be directed by ligand-receptor pairs. These findings may be useful for the vectorization of MPs produced *in vitro*.

CD27^+^ MPs have a weaker functional effect than the previously studied TGFβ^+^ MPs (15), which are also derived from heterologous blood products. They can greatly increase the intensity of the CD27 phenotype of cells already expressing CD27, but their functional benefit remains to be clarified.

The contribution of exogenous CD27^+^ MPs supplied by transfusion should, however, be taken into account in patients treated with immunotherapies based on monoclonal anti-CD27 antibodies. Our *in vitro* assay results indicate that the repeated transfusion of CD27^+^ MPs may result in these particles competing with the cellular targets of this immunotherapy.

## Data availability statement

The original contributions presented in the study are included in the article/**Supplementary Material**. Further inquiries can be directed to the corresponding author.

## Ethics statement

Ethical review and approval was not required for the study on human participants in accordance with the local legislation and institutional requirements. Written informed consent to participate in this study was provided by the participants’ legal guardian/next of kin.

## Author contributions

BV was the principal investigator and takes primary responsibility for the paper. MT, LC, DN-L performed the laboratory work. LC and BV analyzed the results. SC provided platelet concentrates. BV coordinated the research. BV, LC, DN-L and FP wrote the paper. LB and MK reviewed the manuscript. All authors contributed to the article and approved the submitted version.
