# Supplementary material for: CD27+ microparticle interactions and immunoregulation of CD4+ T lymphocytes
Source: Front Immunol. 2023 Mar 9;14:1043255. doi: 10.3389/fimmu.2023.1043255 (PMC10034125; doi:10.3389/fimmu.2023.1043255)
Supplement: Supplementary file 1 [file DataSheet_1.pdf]

CD27^{+} microparticle interactions and immunoregulation of CD4^{+} T lymphocytes

Léonie Cagnet, Deborah Neyrinck-Leglantier, Marie Tamagne, Lylia Berradhia, Mehdi Khelfa, Sabine Cleophax, France Pirenne and Benoît Vingert

Supplemental Information

Supplemental Materials and Methods

Intracellular cytokine staining (ICS) assay

The $CD4^+$ T cells involved in the response to CD27-expressing MPs were evaluated with a LSRFortessa flow cytometer (BD Biosciences, Franklin Lakes, NJ), as previously described. (1) The MPs were resuspended in filter-sterilized medium (passage through a filter with 0.1 μm pores) and the CD27-expressing MPs present in the MP preparation were counted. We then added various amounts of the bulk MP preparation to the culture medium such that we obtained ratios of PBMCs to CD27-expressing MPs of 1:1, 1:10 and 1:20.

The negative controls were PBMCs not cultured with MPs. The positive controls were PBMCs stimulated with the staphylococcal enterotoxin B superantigen (1 μg/ml, List Biological Laboratories, Inc, Campbell, CA).

Cells were harvested and stained for surface antigens with CD4-APC-H7 and CD3-PE antibodies (BD Biosciences, Franklin Lakes, NJ). IFNγ-Pe-Cy7, IL2-BV605, and TNFα-BV711 (Biolegend, San Diego, CA) antibodies were used for the detection of intracellular cytokines.

(1) Coombs J, Ben Hassen L, Leclerc M, Tamagne M, Pannetier L, Khelfa M, et al. Dominant immune response to HLA-B57/B58 molecules after platelet transfusion. Transfusion (2020) 60:2807–2814. doi: 10.1111/trf.16116.

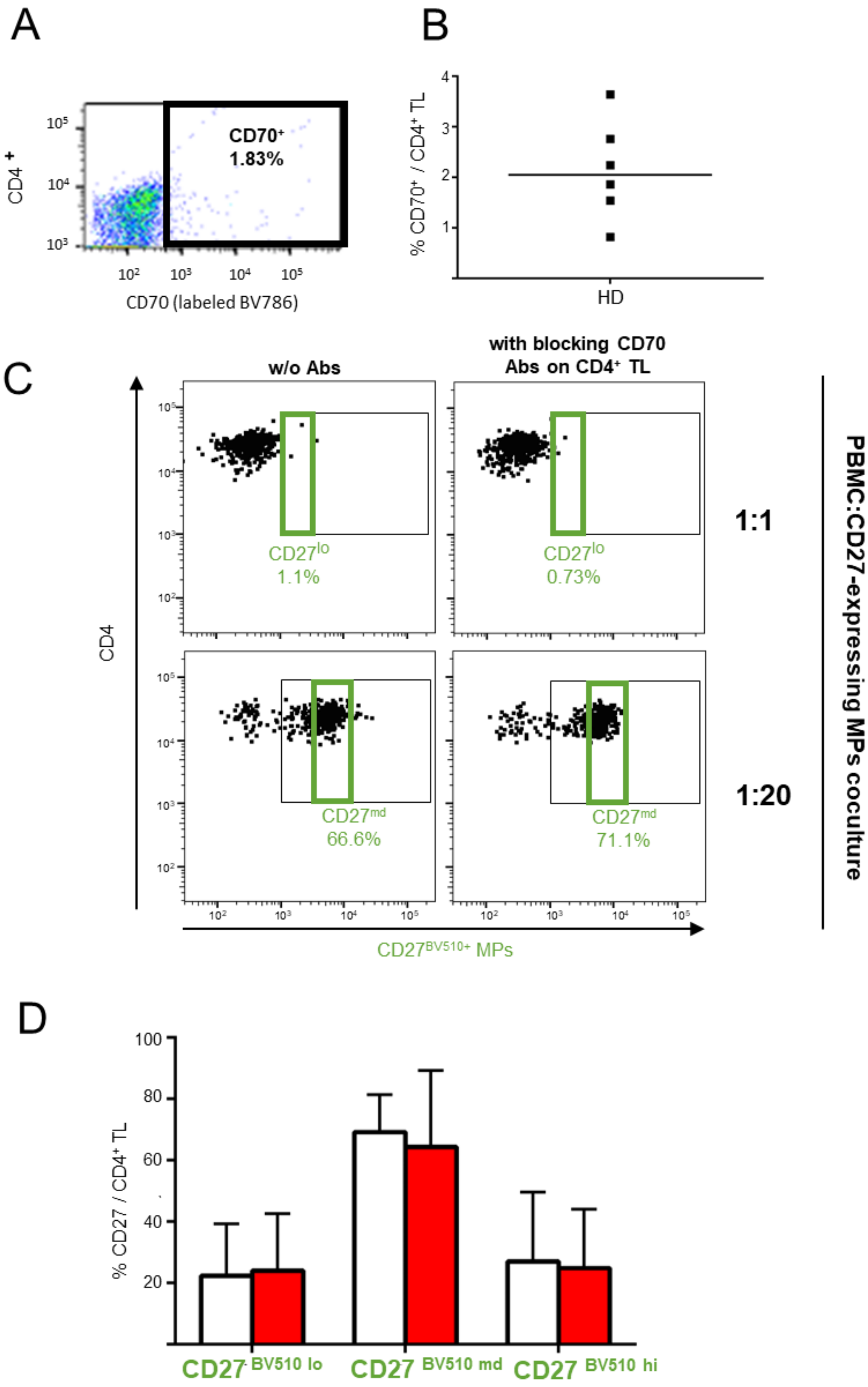
A
B
CD4 +
CD70+
1.83%
CD70 (labeled BV786)
% CD70+ / CD4+ TL
HD
C
w/o Abs
with blocking CD70
Abs on CD4+ TL
CD27lo
1.1%
CD27lo
0.73%
CD27md
66.6%
CD27md
71.1%
1:1
1:20
CD4
CD27BV510+ MPs
PBMC:CD27-expressing MPs coculture
D
% CD27 / CD4+ TL
CD27 BV510 lo
CD27 BV510 md
CD27 BV510 hi

<u>Supplemental Figure 1</u>: $CD27^+$ expressing MP binding to $CD4^+$ TLs after MPs coculture and cellular CD70 labeling. (A) Example of CD70 phenotyping on $CD4^+$ TLs with an anti-CD70 antibody (labeled with BV786). Phenotyping was performed on $CD4^+$ TLs from 25 HDs in two experiments. (B) Phenotyping of the CD70 receptor was performed on $CD4^+$ TLs from six HDs in two experiments. The horizontal bar indicates the median. (C) Example of the gating strategy used to highlight the expression of CD27 from MPs, on the surface of $CD4^+$ TLs (PBMCs were stained with anti-CD4 and anti-CD3 antibodies; $CD4^+$ TLs were defined as having a $CD3^+CD4^+$ phenotype; only $CD4^+$ TLs are presented), in the presence and absence of blocking anti-CD70 antibody. PBMCs were cultured with various amounts of the bulk MP preparation to the culture medium such that we obtained ratios of PBMCs to CD27-expressing MPs of 1:1, 1:10 and 1:20. (D) PBMCs labeled with CD70 antibody were cocultured for 18 hours with known numbers of CD27-expressing MPs at ratios of 1:1, 1:10 and 1:20. Cocultures were assessed for seven HDs in four experiments. The percentage of $CD4^+CD27^{lo}$ TLs was determined for the 1:1 ratio (PBMCs: CD27-expressing MPs); the percentage of $CD4^+CD27^{md}$ TLs was determined for the 1:10 ratio, and the percentage of $CD4^+CD27^{hi}$ TLs was determined for the 1:20 ratio, with (■) and without (□) blocking anti-CD70 antibody.

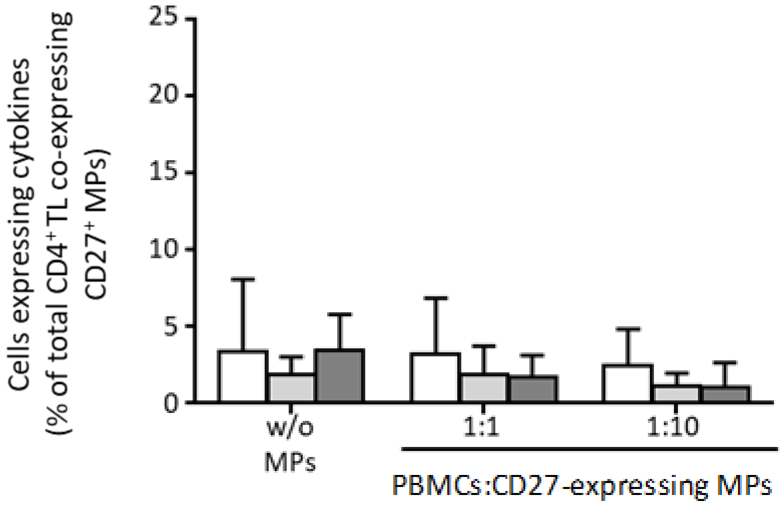

<u>Supplemental Figure 2</u>: Cytokine expression by CD4$^+$ T cells after coculture with CD27-expressing MPs. PBMCs were cultured with various amounts of the bulk MP preparation to the culture medium such that we obtained ratios of PBMCs to CD27-expressing MPs of 1:1, 1:10 and 1:20. Cocultures were assessed for PBMCs from 12 HDs in four experiments. Cytokine expression was assessed for CD4$^+$ TLs in an intracellular cytokine staining (ICS) assay. Three cytokines were studied, IL2 (□), TNFα (■) and IFNγ (■).
